# A New Perspective on the Management of Giant Rectal Polyps Presenting With McKittrick-Wheelock Syndrome: A Case Report on Treatment With Transanal Excision and Delorme’s Plication

**DOI:** 10.7759/cureus.80075

**Published:** 2025-03-05

**Authors:** Andrew C Ekwesianya, Abraham V Jesudoss, Manoj Jacob, Mohamad F Badr, Bandipalyam V Praveen

**Affiliations:** 1 Department of General and Colorectal Surgery, Southend University Hospital, Southend, GBR

**Keywords:** acute kidney injury, colorectal polyps, electrolyte imbalance, mckittrick-wheelock syndrome, villous adenoma

## Abstract

McKittrick-Wheelock syndrome is characterized by fluid deficit, electrolyte derangements, and acute kidney injury caused by a villous colorectal polyp. Treatment depends on both the location and size of the polyp. Most small polyps are treated by endoscopic resection and larger polyps by either surgical segmental resection or endoscopic excision. The most challenging polyps are those involving the lower rectum, often associated with rectal prolapse. Endoscopic excision is much more difficult, and surgical resection remains the predominant treatment option. We discuss an 83-year-old female with a large low rectal polyp who is presenting with "chronic diarrhea," electrolyte deficiencies, acute-on-chronic kidney disease, hypoalbuminemia, and anemia. Clinical assessment, colonoscopy, radiological imaging, and histological diagnosis were consistent with a large tubulovillous adenoma with low-grade dysplasia. After physiological optimization, the lesion was successfully treated by transanal submucosal excision and Delorme’s plication, instead of an abdominoperineal resection; there was a prompt resolution of the symptoms. A patient with the triad of chronic diarrhea, acute renal insufficiency, and electrolyte deficiencies should have a colonoscopy to rule out a villous colorectal polyp. Low rectal polyps that are too large to be resected endoscopically could benefit from transanal excision and Delorme’s plication, instead of an abdominoperineal resection.

## Introduction

Recurrent electrolyte imbalance and significant fluid loss from a colorectal mucinous polyp have been reported in medical literature for decades. McKittrick and Wheelock in 1954 described a case of villous adenoma of the rectum presenting with electrolyte imbalance and acute kidney injury [1]. These polyps typically have finger-like projections that secrete large amounts of fluid and mucin, resulting in loss of fluids, electrolytes, and protein. Acute kidney injury can result if the fluids are not adequately replaced [2]. Very low rectal polyps can prolapse through the anal verge and become subject to trauma and frequent bleeds.

Most benign colorectal polyps are treated by endoscopic resection. In the case of a villous polyp that involves a large surface of the rectum or colon, however, a large defect is left after excision; as a result, segmental surgical resection is frequently performed.

In this article, we discuss a case of a large low rectal villous adenomatous polyp that presented with features of McKittrick-Wheelock syndrome at Southend University Hospital, Westcliff-on-Sea, England. The aim is to highlight the extent of fluid-electrolyte loss, malnutrition, and anemia that could result from this polyp if treatment is delayed, and to discuss the method of rectal wall closure following a transanal excision of a large polyp.

## Case presentation

Clinical assessment

An 83-year-old female was referred to the pelvic floor clinic with recurrent bright-red rectal bleeding and copious discharge of mucus per rectum described as "diarrhea," for three years. She subsequently experienced reducible prolapse of a fleshy mass from the anal verge. There was no weight loss or rectal pain. She had been admitted multiple times in the past and managed for "chronic diarrhea," with recurrent hyponatremia, hypokalemia, anemia, and progressively deteriorating renal function. She had also been referred to the pelvic floor colorectal clinic but her treatment was delayed due to the Covid-19 pandemic.

The patient’s medical history included hypertension, atrial fibrillation, chronic kidney disease, ischemic heart disease, and dysphagia.

Physical examination revealed chronic excoriation of the perianal skin, increased pelvic descent on straining suggestive of underlying weak pelvic floor muscles, reduced anal resting and squeeze pressures, and a palpable large rectal polyp. A clinical diagnosis of rectal polyp with rectal prolapse was made.

Investigations

**Figure 1:**
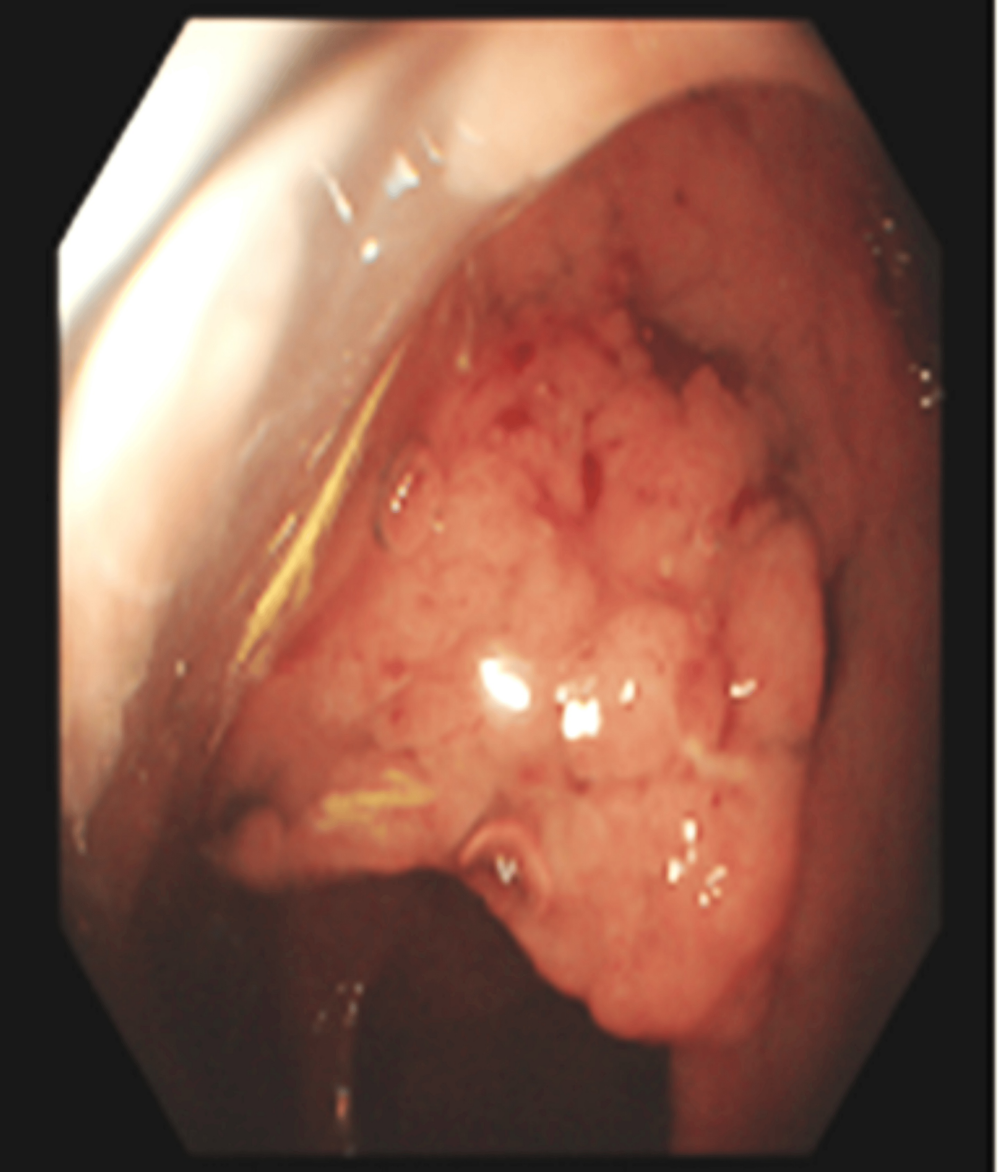
The rectal polyp as seen on colonoscopy

The patient underwent a colonoscopy, which revealed a rectal polyp (likely a tubulovillous adenoma) in contact with the dentate line, not amenable to endoscopic resection. Fourth-degree hemorrhoids were also found. A colonoscopic image of the lesion is shown in Figure 1.

Histology of the biopsy specimens revealed a large intestinal mucosa showing pieces of a tubulovillous adenoma with low-grade dysplasia.

**Figure 2:**
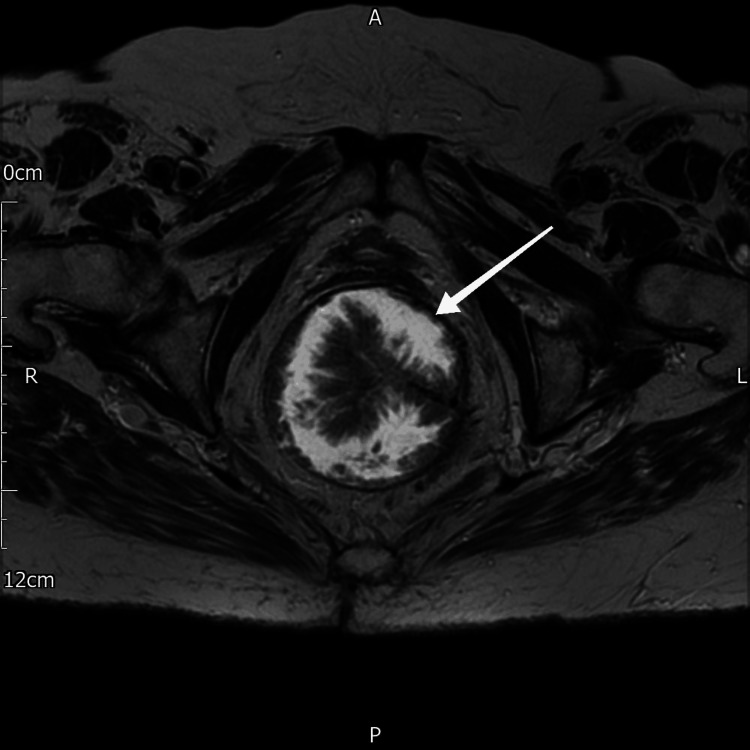
MRI image of the rectal polyp The white arrow indicates the tumor, with finger-like villi projecting into the rectal lumen.

CT of the chest, abdomen, and pelvis reported a rectal mass in keeping with a malignancy; no obvious nodal or metastatic disease to the extent visualized. Further assessment of the lesion was conducted with an MRI of the pelvis and rectum, which revealed a large venous-appearing mass measuring approximately 8 cm in the lower mid-rectum, with the base of the lesion along the left wall. There was a suspicious signal at the level of the anorectal junction, approximately 3 cm above the anal verge, between the 1 o'clock and 3 o'clock positions, which appeared to involve the left levator, suspicious of malignancy. A large volume of mucin was seen, but no definite abnormal lymphadenopathy was noted. No convincing evidence of extra-mucosal vascular invasion was observed. The MRI image of the lesion is shown in Figure 2.

Treatment

The patient was discussed in the colorectal cancer multidisciplinary (MDT) meeting and the consensus recommendation was that the lesion was a benign polyp but the patient should have surgery for symptom relief.

**Figure 3:**
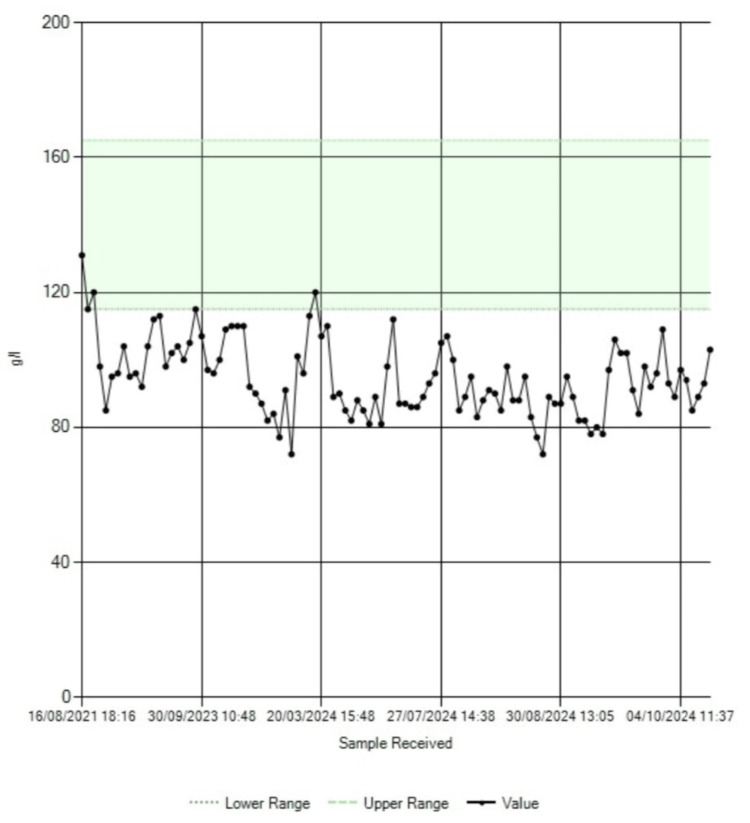
Trend in blood hemoglobin levels

**Figure 4:**
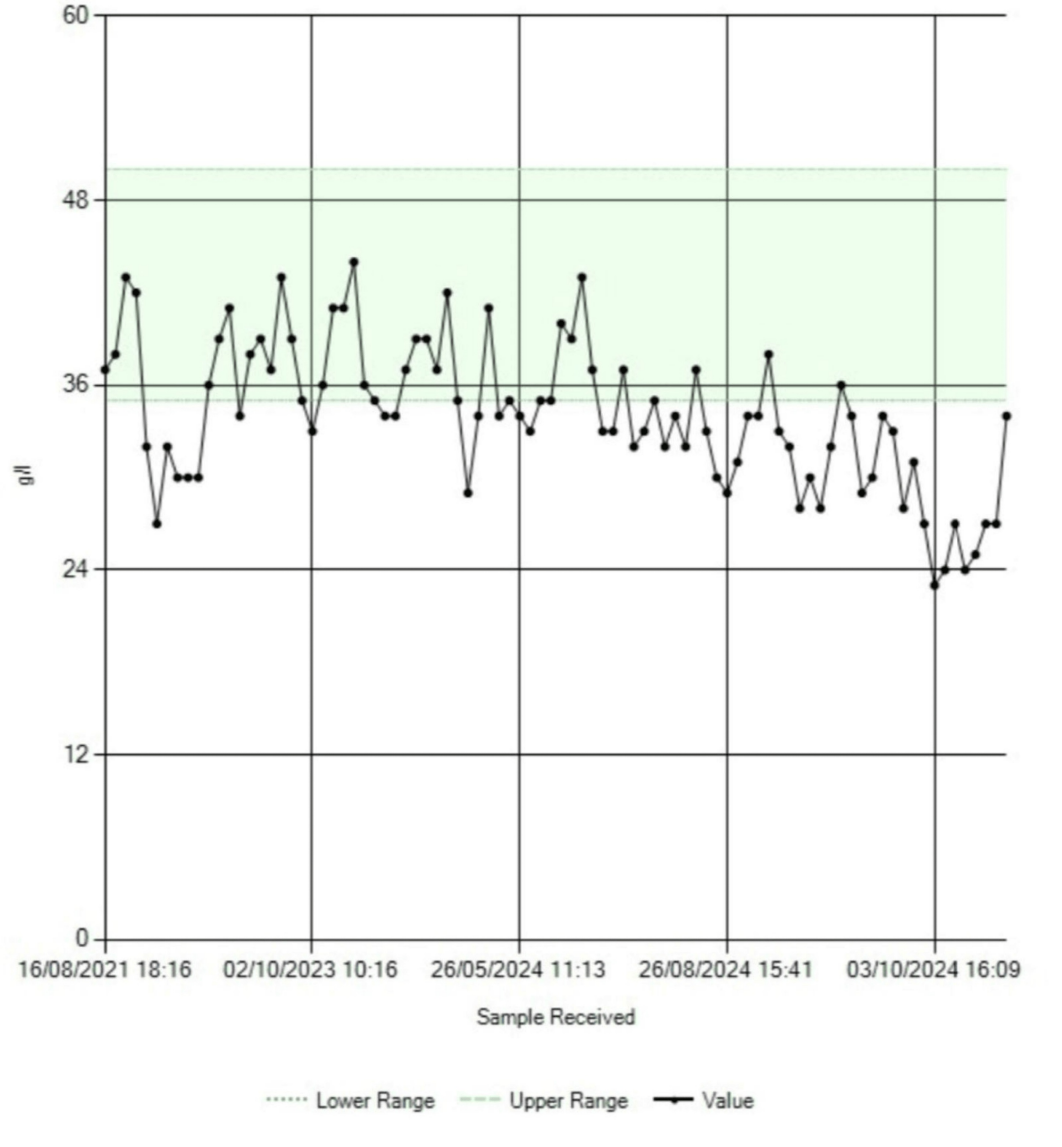
Trend in serum albumin levels. Note the increase in albumin levels toward normal following surgery in October 2024

**Figure 5:**
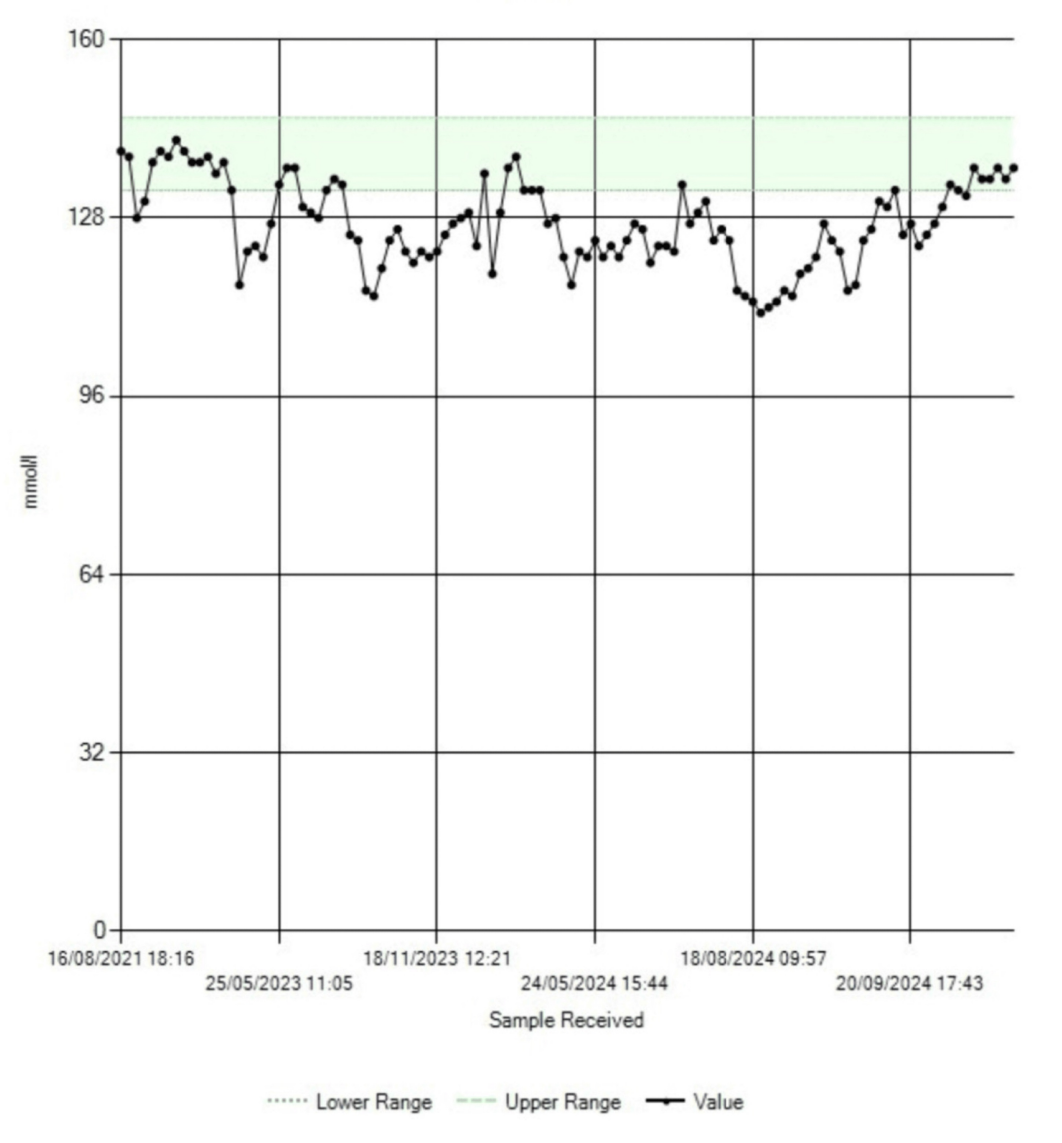
Trend in serum sodium levels. Notably, serum sodium returned to the reference range after surgery

**Figure 6:**
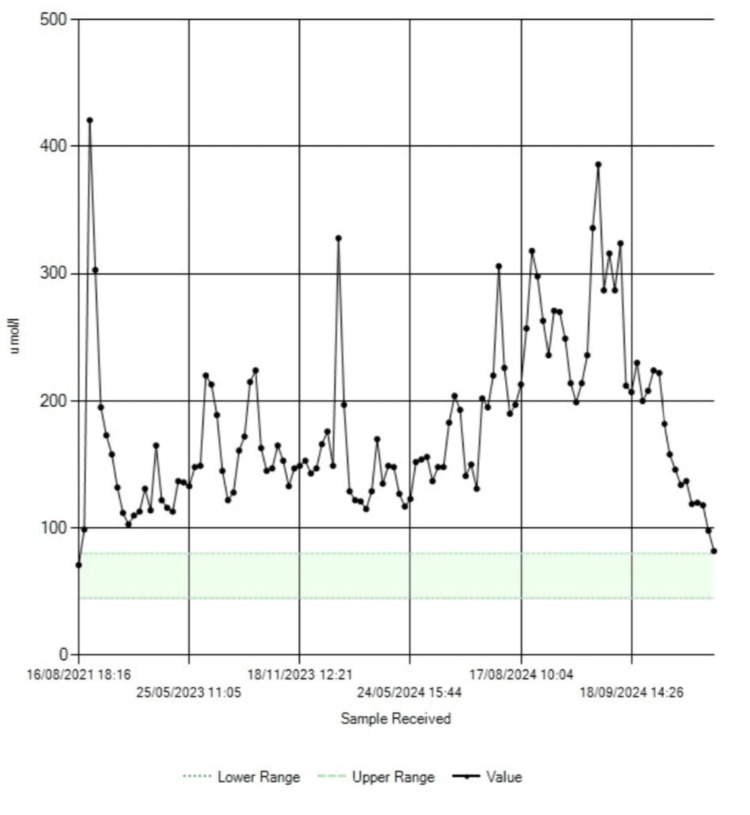
Trend in serum creatinine levels. Notably, there is a sharp decline in serum creatinine following surgery

While awaiting outpatient anesthetic review, the patient was readmitted through the emergency department with worsening anemia, electrolyte imbalance, and acute-on-chronic kidney disease. The trends in the blood hemoglobin, serum albumin, serum sodium, and serum creatinine levels are shown in Figure 3, Figure 4, Figure 5, and Figure 6, respectively.

She was admitted under the joint care of colorectal surgeons and nephrologists to optimize her multiple co-morbidities. Preoperative optimization included intravenous fluids, electrolyte replacement, parenteral nutrition, and red blood cell transfusion. She subsequently had an urgent surgery in October 2024, as an inpatient.

**Figure 7:**
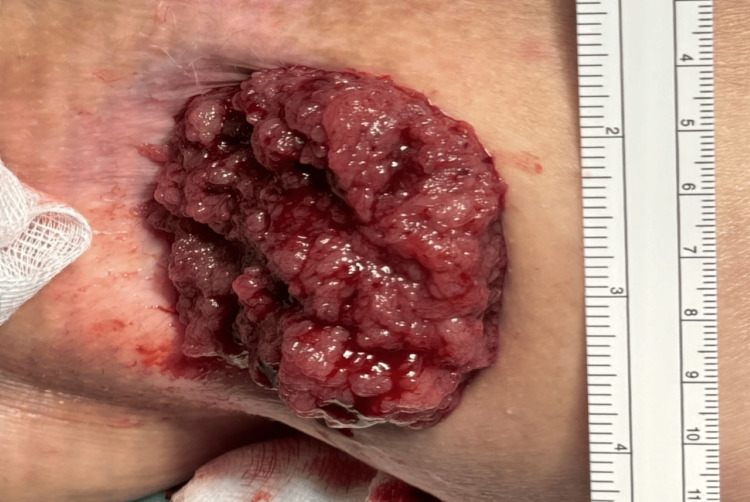
Intraoperative picture of the rectal polyp

Examination under anesthesia revealed a cauliflower-like distal rectal polyp extending longitudinally from the anorectal junction to approximately 8 cm proximally, and transversely from the 2 o'clock to 10 o'clock positions, causing rectal mucosal prolapse. There were copious amounts of mucin in the rectum. The intraoperative picture of the lesion is shown in Figure 7.

**Figure 8:**
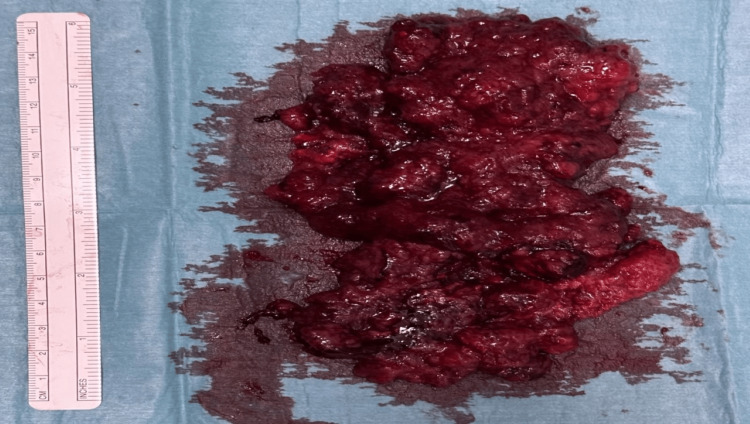
A picture of the polyp after resection

**Figure 9:**
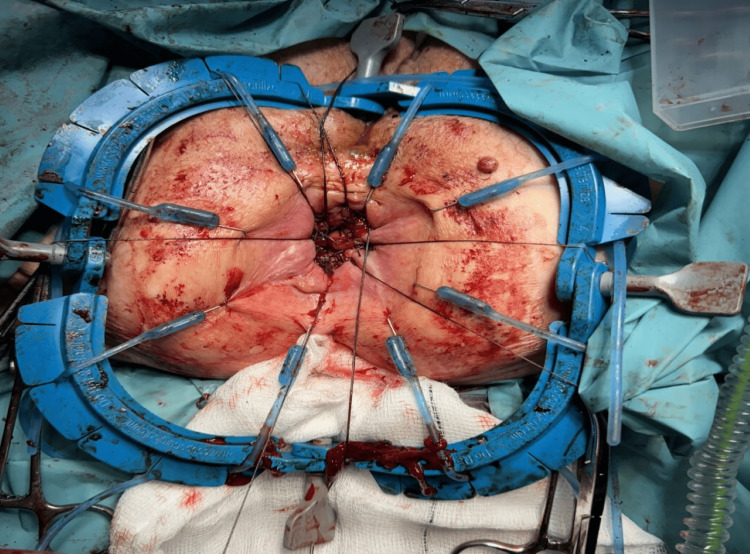
Intraoperative picture of the anal canal after resection of the polyp and Delorme’s plication

A transanal submucosal excision of the polyp with Delorme’s plication was performed. The resected specimen is shown in Figure 8, and the immediate post-operative outlook of the anal canal is shown in Figure 9.

**Figure 10:**
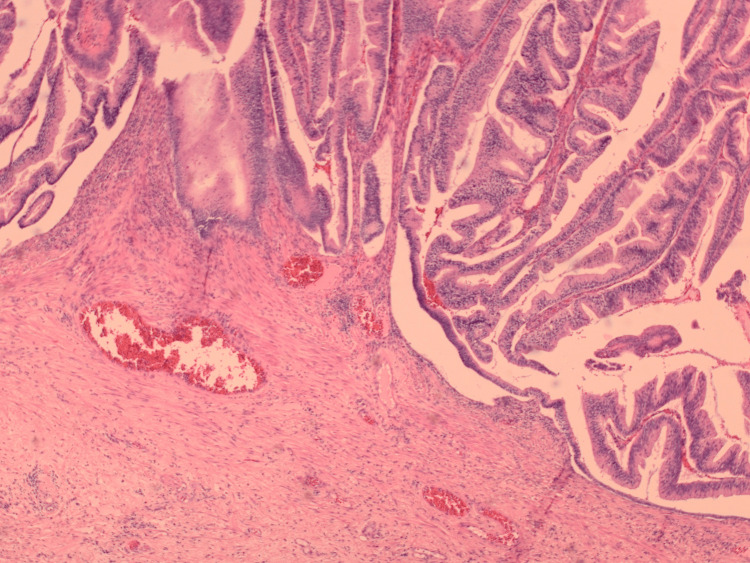
Histological image of a rectal polyp showing typical villiform structures with abnormal cell covering. Importantly, the junction with the normal supporting tissue (pink collagen) is well-defined, indicating no invasion. Staining: Haematoxylin and Eosin. Magnification: ×20 (using a ×2 lens with a ×10 objective)

The final histology report of the resected specimen showed a rectal Delorme's specimen measuring 170 mm x 85 mm x 10 mm, containing an exophytic polyp measuring 85 mm x 40 mm x 50 mm, representing a very large tubulovillous adenoma with low-grade dysplasia and areas of serrated architecture. Careful scrutiny, importantly, found no definite invasion. There were small areas of normal squamous epithelium distally, suggesting parts of the anal canal, and this margin was uninvolved by the polyp. A representative low-power image of the histology of the adenoma is shown in Figure 10.

The patient had an unremarkable post-operative recovery and was medically fit for discharge five days after the surgery. Three months postoperatively, she was doing well with no sign of local recurrence. Her bowel function was regular with good control; her quality of life had significantly improved allowing her to be independent. Her renal parameters and electrolyte disturbances had also improved.

## Discussion

Adenomas that cause McKittrick-Wheelock syndrome are the ones located in the rectum or sigmoid and large enough (>3-4 cm) to produce symptoms [3]. They secrete sodium and potassium, and the sodium gradient leads to secretory diarrhea. The distal location of the tumor does not allow an adequate compensatory reabsorption mechanism, given the absence of normal colonic mucosa distally [4,5].

In addition to renal insufficiency, symptoms of this syndrome could also be related to electrolyte depletion, including vomiting, confusion, asthenia, and cardiac arrhythmias. Patients with an unrecognized long-term syndrome may need hemodialysis or be at risk of life-threatening events at the time of presentation. A triad of chronic diarrhea, pre-renal acute kidney injury, and electrolyte imbalance should, therefore, prompt the search for a colorectal villous adenoma [6].

Large villous colorectal polyps that present with features of McKittrick-Wheelock syndrome pose both medical and surgical treatment challenges. Preoperative optimization includes correction of fluid and electrolyte derangements, anemia, hypoproteinemia, and renal dysfunction. In addition, preoperative endoscopic and radiological investigations are necessary for accurate histological diagnosis and exclusion of a malignant polyp. Surgical treatment depends on both the location and size of the polyps. Most small colonic polyps are treated by endoscopic resection and larger polyps by either surgical segmental resection or endoscopic piecemeal excision.

In a systematic literature review of 257 cases of colorectal polyps with McKittrick-Wheelock syndrome, Orchard and colleagues reported that the majority (64.8%) of the polyps were treated by anterior resection, abdominoperineal resection, or sigmoid colectomy with or without colostomy. Only about 5.4% of the patients were treated by transanal endoscopic resection, some of whom later required surgical resection or repeat endoscopic procedures [7].

Large rectal polyps that involve a wide surface area of the rectum are much more challenging to treat by minimal access approach, due to problems with recurrence and the large rectal mucosal defect left after the excision. As a result, they have been traditionally treated by low anterior resection or abdominoperineal resection [8,9]. In 2024, Kouladouros et al. reported successful treatment of a giant (19x10 cm) rectal polyp excised by endoscopic submucosal resection [9].

The most challenging polyps, however, are those involving the lower rectum, often associated with rectal prolapse. Endoscopic excision of these polyps is much more difficult, and surgical resection remains the predominant treatment option [10]. In frail elderly patients in whom the risk of such an extensive surgical procedure is prohibitive, a less radical treatment option is necessary.

In our 83-year-old patient with a large low rectal polyp, rectal mucosal prolapse, and multiple significant co-morbidities, we performed a transanal submucosal excision of the tumor, and the large rectal mucosal defect was closed by Delorme’s plication. This procedure not only addressed the large defect created by the excision of the tumor but also the rectal prolapse caused by the polyp.

## Conclusions

McKittrick-Wheelock syndrome is an entity that colorectal surgeons, gastroenterologists, and nephrologists should always consider as a differential diagnosis in patients who have the triad of chronic diarrhea, acute kidney injury, and electrolyte deficiencies. This would help obtain a prompt diagnosis and an appropriate treatment. Prompt treatment would resolve a condition that may develop life-threatening complications. In addition, if left untreated for a long time, the adenoma could potentially evolve into an invasive cancer.

Large villous rectal adenomas that present with McKittrick-Wheelock syndrome pose specific medical and surgical treatment challenges. They are mostly treated by surgical resection and less commonly by transanal endoscopic excision. In this article, however, we described a large low rectal polyp in an elderly patient that was successfully treated by transanal submucosal resection and Delorme’s plication.

## References

[1] McKittrick LS, Wheelock FC (1997). Carcinoma of the colon. Dis Colon Rectum.

[2] Peress S, Bonilla C, Yoo ER, Brinkerhoff B, Bakis G, Yu J (2024). McKittrick-Wheelock syndrome: chronic diarrhea and electrolyte abnormalities due to a large rectal polyp with endoscopic management. Gastrointest Endosc.

[3] Falkinham JO 3rd (1979). Gene lon and plasmid inheritance in Escherichia coli K-12. J Bacteriol.

[4] Lee YS, Lin HJ, Chen KT (2012). McKittrick-Wheelock syndrome: a rare cause of life-threatening electrolyte disturbances and volume depletion. J Emerg Med.

[5] Older J, Older P, Colker J, Brown R (1999). Secretory villous adenomas that cause depletion syndrome. Arch Intern Med.

[6] Emrich J, Niemeyer C (2002). The secreting villous adenoma as a rare cause of acute renal failure. Med Klin (Munich).

[7] Orchard MR, Hooper J, Wright JA, McCarthy K (2018). A systematic review of McKittrick-Wheelock syndrome. Ann R Coll Surg Engl.

[8] de Sousa Miranda I, Ferreira JR, Rocha S, Monteiro M, Guillerme J, Domingos R (2022). McKittrick-Wheelock syndrome: a neoplastic cause of electrolyte imbalance. Eur J Case Rep Intern Med.

[9] Kouladouros K, Schneider K, Kubicka S, Hoerner C, Hirth M (2024). Endoscopic submucosal dissection of a giant rectal adenoma manifesting as McKittrick-Wheelock syndrome. Z Gastroenterol.

[10] Villanueva ME, Onglao MA, Tampo MM, Lopez MP (2022). McKittrick-Wheelock syndrome: a case series. Ann Coloproctol.

